# Are We Right on Target? Is Comprehensive Genomic Profiling Ready for Prime Time in Resource-Constrained Settings?

**DOI:** 10.1200/GO.22.00135

**Published:** 2022-06-17

**Authors:** Joydeep Ghosh, Gilberto Lopes, Surpiya Chopra

**Affiliations:** ^1^ Tata Medical Center, New Town, India; ^2^ Sylvester Comprehensive Cancer Center, Miami, FL; ^3^ Tata Medical Center, Mumbai, India

Comprehensive genomic profiling (CGP) uses next-generation sequencing methods to detect specific molecular abnormalities in a patient's tumor. CGP can disclose biomarkers at nucleotide-level resolution and typically comprises all major genomic variant classes, as well as large genomic signatures, maximizing the ability to find clinically actionable alterations.^1^

There are several reasons why CGP is becoming commonly used in higher-resource settings. First, almost all advanced malignancies evolve and become resistant to standard chemotherapy after initial treatment. As such, there is a need to identify more specific, actionable targets for therapies that we hope might have better efficacy and longer duration of effect. Second, when compared with older chemotherapy agents, most new targeted therapies tend to have less severe toxicity. This allows for patients with poorer performance status to receive therapy that might otherwise be prohibitive with chemotherapy. Finally, CGP-driven medicine has the potential to make the treatment of resistance more rational and actionable.

Many investigators have tried to do widespread genomic profiling in various tumor subtypes. The Cancer Genome Atlas has made efforts to sequence thousands of cancer cell lines. The most common mutations identified were von Hippel Lindau (*VHL*), *TP53*, phosphatidylinositol-4,5-bisphosphate 3-kinase catalytic subunit alpha (*PIK3CA*), phosphatase and tensin (*PTEN*) homolog deleted on chromosome 10, phosphoinositide-3-kinase regulatory subunit 1, Kirsten rat sarcoma (*KRAS*) viral oncogene homolog, and adenomatous polyposis coli genes (*FAP*).^2^

Many specific targeted therapies have achieved significant clinical success, such as oral tyrosine kinase inhibitors in chronic myelogenous leukemia, lung cancer, breast cancer, and colorectal and renal cell carcinoma.^3–7^ That notwithstanding, not all trials have shown positive results with specific targets and therapies in broader settings. An example is the SHIVA trial, where molecular alterations were identified within one of three molecular pathways (hormone receptor, phosphatidylinositol 3-kinase/protein kinase B or Akt/mammalian target of rapamycin, and *Raf* gene/mitogen activated protein kinase), which could be matched to one of 10 targeted regimens. One-hundred ninety-five patients were randomly assigned. There were no differences in progression-free survival compared with standard of care. This trial informs us that broadly molecular-driven therapies may not always be more effective than older therapies.

In lower-resource settings, the availability of specific therapies is an even bigger issue. Although there are significant challenges in access to cancer treatment, that should not preclude concurrent deployment of beneficial newer therapies. In the article that accompanies this editorial by Mathew et al^8^ looked at the results of CGP in a sample of Indian patients living with cancer. This is one of the first-of-its-kind studies from India and in lower-middle-income countries, and we congratulate the authors for that. They conducted a retrospective cohort study among patients who underwent CGP for advanced cancers in the South Asian nation. Patients received therapy in different lines of therapy, and various platforms were used to search for targets. Therapy was decided on the basis of the presence of a targetable mutation at the discretion of the treating oncologist. The primary end point was to assess the proportion of patients who were eligible for targeted therapy. The secondary objective was clinical benefit. Patients who had approved or accepted therapies for their respective cancers, for example, epithelial growth factor receptor (*EGFR*) mutation in lung or human epidermal growth factor receptor 2 (HER2)–directed therapy in the breast, were excluded. A total of 221 patients were included. The majority of patients underwent tissue biopsy (90%). The most common cancers were lung (18%), breast (15%), pancreaticobiliary (9%), and colorectal (7%). Out of 96 patients who had a targetable mutation, only 21 (10%) actually received specific therapy. The most common reason for not receiving specific therapy was standard-of-care treatment, poor performance status, and nonavailability of the drug in India. Among drugs that were not available, 33% (n = 7) were for HER2 amplification (nonbreast cancer) and 19% for HER2 or *EGFR* exon 20 insertion mutation (lung cancer; n = 4). *EGFR* mutations were seen in two patients. After excluding patients with HER2 or *EGFR* exon 20 insertions (four patients), which are emerging targets in lung cancer, 17 patients out of 217 (8%) received targeted therapy. Clinical benefit (defined as treatment for more than 6 months) was seen in nine patients (4%), of whom two were exceptional responders, receiving the drug for more than 12 months.

This is a timely study, as it brings out the scope of CGP-driven targeted therapy from India for the first time. It also involves multiple centers, so that a wider variety of patients was included. CGP was done in standardized testing centers. However, this was a retrospective study, where the inclusion of patients was subjected to the inherent biases of retrospective studies. Moreover, this was a multicenter study, and the data collection formats may not have been uniform for all the centers. Only a very small percentage of patients (10%) actually received CGP-driven therapy. This is similar to other studies such as the MOSCATO study, conducted at the Gustave Roussy Institute, France, where only 7% benefitted from this strategy.^9^ Despite doing the test, the standard of care was available in 21% and poor performance status in 18%. As such, for almost 40% of the patients, the results did not have actual clinical implications. Moreover, data came from multiple different platforms with a wide variety of gene combinations, making interpretation more challenging.

Performing CGP on a regular basis in India in particular and in LMICs in general has its own challenges. First, and maybe most importantly, testing is not widely available, usually because of cost and lack of expertise. Most of the centers that participated in this study are in major urban areas. Indeed, CGP in this study was most often performed at a center away from where each patient was treated. Even where tests are available, the turnaround time of 3-4 weeks can lead to delays in treatment. Furthermore, the costs of these tests ($200-$1,500 US dollars) and of the corresponding targeted therapies are a major hindrance in the utilization and implementation of such an approach. This is clearly reflected in the results of this study, in which the overwhelming majority of patients did not receive targeted therapy. Although the results may be interpreted as a lack of benefit, they are also indicative of the lack of accessibility of newer agents in the country. The National Cancer Grid of India guidelines do not presently enlist a majority of targeted therapies as essential. Furthermore, the quantum of reimbursement received by private insurance (range, $4,000-$15,000 US dollars/financial year) practically means that a majority of patients who go ahead with targeted therapies will incur out-of-pocket expenditure. There is therefore a clear need to advocate for lower pricing of targeted therapies that are identified to be of significant clinical benefit in various cancers to avoid a health care implementation divide between high-income and low-income and lower-middle-income countries.

Are we ready for CGP and targeted therapy in low-income or lower-middle-income countries? The answer, at current cost and lack of availability, is still a resounding NO. We need to address many of the aspects we mentioned in this editorial. There has to be better access to newer targeted agents and to clinical trials. More and more academic centers should enlist themselves as sponsored or investigator-initiated trial centers. There should be wider access to newer molecules. Most of the drugs that get approved by Western regulatory authorities take a significant amount of time to be approved in India because of the stringent rules laid down by Indian regulatory authorities. Although this may be beneficial to ensure that medications have clear benefits, it may also delay or deny access to useful treatments. Differential pricing and increased insurance coverage, for instance, should be considered by both the pharmaceutical industry and insurance providers.

Separate from the challenges with industry, there is also a lack of proper understanding of the utility of clinical trials among patients and their caregivers. That also needs to be addressed with proper awareness programs on a wider scale. We also need to determine the most relevant and precise set of genes and tests in each clinical situation that is actually helpful and may be targeted by available therapies. This would bring down cost and turnaround time. As clinicians, we need to identify a subset of patients to whom we can apply CGP. This should take into account the performance status, affordability, availability of cheaper alternatives, disease status, and long-term outcomes with targeted therapies. This should prevent blanket testing for a large majority of patients.

There is a need to have better patient access programs and phase IV studies in low- and middle-income countries as well as participation in clinical trials in general of more diverse populations than the ones currently included in trials. Although patients from East Asia seem to be increasingly enrolled in clinical trials, most studies are still predominantly run in the United States and Western Europe, with little involvement from Latin America and South Asia and virtually none from sub-Saharan Africa, with the exception of South Africa. It is indeed ironic that for cancers that are common in India (eg, cervical cancer), very limited targeted agents or immunotherapy studies have representation of Indian patients. While these strategies may be convenient at an early stage for pharmaceutical companies, in the long run, however, it may be counterproductive for their economics, since high-incidence regions will continue not to list drugs, such as bevacizumab and pembrolizumab, as essential for lack of either safety, population-specific data, or cost-benefit analysis. The pharmaceutical industry, therefore, needs to blend the search for profit with social responsibility for the long-term sustainability of their drugs for cancers that are common in low- and middle-income countries. We sincerely hope that while chasing targeted therapy, we do not miss the right target.

Despite all the hurdles, a large number of patients, especially those who are having reimbursement schemes from government or private insurance companies, get the optimal target-driven treatment and derive the benefit. Although the fraction is small compared with the cancer burden, it is a sign of feasibility, especially with the help of administrative changes and better awareness of benefits with targeted therapies.
