# Erratum

**DOI:** 10.1200/GO.22.00249

**Published:** 2022-09-21

The article by Ghosh et al entitled “Are We Right on Target? Is Comprehensive Genomic Profiling Ready for Prime Time in Resource-Constrained Settings?” (*JCO Global Oncol* 10.1200/GO.22.00135) was published June 17, 2022 with errors.

The third author's name and affiliation incorrectly read in the author list, author's disclosures of potential conflicts of interest section, as:

Surpiya Chopra, MD, DNB^3^

^3^Tata Medical Center, Mumbai, India

The name and affiliation should have read as:

**Supriya** Chopra, MD, DNB^3^

^3^Tata Me**morial Center**, Mumbai, India

This has been corrected as of September 21, 2022. The authors apologize for the errors.

